# Structural basis of chitin utilization by a GH20 β-*N*-acetylglucosaminidase from *Vibrio campbellii* strain ATCC BAA-1116

**DOI:** 10.1107/S2059798321002771

**Published:** 2021-04-27

**Authors:** Piyanat Meekrathok, Marco Bürger, Arthur T. Porfetye, Sawitree Kumsaoad, Anuwat Aunkham, Ingrid R. Vetter, Wipa Suginta

**Affiliations:** a Biochemistry–Electrochemistry Research Unit, Suranaree University of Technology, Nakhon Ratchasima 30000, Thailand; b Max-Planck Institute of Molecular Physiology, 44227 Dortmund, Germany; c School of Biomolecular Science and Engineering (BSE), Vidyasirimedhi Institute of Science and Technology (VISTEC), 555 Payupnai, Wangchan, Rayong 21210, Thailand

**Keywords:** GH20 β-*N*-acetylglucosaminidase, chitin recycling, *Vibrio* spp., marine bacteria

## Abstract

*Vibrio* species play a crucial role in maintaining the carbon and nitrogen balance between the oceans and the land through their ability to employ chitin as a sole source of energy. This study describes the structural basis for the action of the GH20 β-*N*-acetylglucosaminidase (*Vh*GlcNAcase) in chitin metabolism by *Vibrio campbellii* (formerly *V. harveyi*) strain ATCC BAA-1116. Crystal structures of wild-type *Vh*GlcNAcase in the absence and presence of the sugar ligand, and of the unliganded D437A mutant, were determined. *Vh*GlcNAcase contains three distinct domains: an N-terminal carbohydrate-binding domain linked to a small α+β domain and a C-terminal (β/α)_8_ catalytic domain. The active site of *Vh*GlcNAcase has a narrow, shallow pocket that is suitable for accommodating a small chitooligosaccharide. *Vh*GlcNAcase is a monomeric enzyme of 74 kDa, but its crystal structures show two molecules of enzyme per asymmetric unit, in which Gln16 at the dimeric interface of the first molecule partially blocks the entrance to the active site of the neighboring molecule. The GlcNAc unit observed in subsite −1 makes exclusive hydrogen bonds to the conserved residues Arg274, Tyr530, Asp532 and Glu584, while Trp487, Trp546, Trp582 and Trp505 form a hydrophobic wall around the −1 GlcNAc. The catalytic mutants D437A/N and E438A/Q exhibited a drastic loss of GlcNAcase activity, confirming the catalytic role of the acidic pair (Asp437–Glu438).

## Abstract 2

Crystal structures of a GH20 β-*N*-acetylglucosaminidase from *V. campbellii* reveal substrate specificity in chitin utilization.

## 1. Introduction   

In marine environments, homeostasis of chitin degradation and biosynthesis is largely maintained by marine *Vibrio* species, and the rate of chitin turnover in oceans is determined by the growth rate of these bacteria (Yu *et al.*, 1991 ▸; Bassler *et al.*, 1991 ▸; Keyhani & Roseman, 1999 ▸; Park *et al.*, 2000 ▸). *V. campbellii* is a bioluminescent member of the marine *Vibrio* species that contributes significantly to the chitin-recycling process, since it can grow extremely quickly under aerobic or anaerobic conditions and strictly requires chitinous materials as its sole source of energy (Suginta *et al.*, 2004 ▸, 2010 ▸; Soto-Gil & Zyskind, 1984 ▸). We previously demonstrated that *V. campbellii* strain ATCC BAA-1116, which has recently been reclassified by the NCBI as *V. campbellii* strain ATCC BAA-1116 (https://www.ncbi.nlm.nih.gov/Taxonomy/Browser/wwwtax.cgi?id=338187), has highly active chitin-utilization machinery (Suginta *et al.*, 2004 ▸; Suginta, Chumjan, Mahendaran, Schulte *et al.*, 2013 ▸; Suginta, Chumjan, Mahendaran, Janning *et al.*, 2013 ▸). Although the chitin catabolic cascade has been proposed to involve a complex series of enzymatic actions, incorporating additional specific chitooligosaccharide/GlcNAc transporters and chitin-binding proteins (Suginta, Chumjan, Mahendaran, Janning *et al.*, 2013 ▸; Li & Roseman, 2004 ▸), the mechanistic details of the cooperation between the molecular components in the chitin catabolic pathway of this bacterium remain unclear. We have previously described the structure and function of *V. campbellii* (formerly *V. harveyi*) endochitinase A (*Vh*ChiA), the secreted chitinolytic enzyme that is mainly responsible for degrading chitin polysaccharide to chitooligosaccharide fragments (Suginta *et al.*, 2004 ▸, 2005 ▸; Songsiriritthigul *et al.*, 2008 ▸). These chitin oligosaccharides are then transported into the cells through chitoporin (Suginta, Chumjan, Mahendaran, Schulte *et al.*, 2013 ▸; Suginta, Chumjan, Mahendaran, Janning *et al.*, 2013 ▸) and are degraded within the periplasm by uncharacterized exo-β-*N*-acetylglucosamin­idases into monomeric or dimeric GlcNAc molecules, which are then transported through the inner membrane by GlcNAc-specific ABC or (GlcNAc)_2_-specific phosphotransferase-system (PTS) transporters (Suginta *et al.*, 2010 ▸; Suginta, Chumjan, Mahendaran, Janning *et al.*, 2013 ▸; Meekrathok & Suginta, 2016 ▸). These GlcNAc and (GlcNAc)_2_ moieties are subsequently converted to carbon and nitrogen intermediates for the bacterium to use as its energy source.

In this study, we focus on a β-*N*-acetylglucosaminidase (*Vh*GlcNAcase) that is a member of the GH20 glycoside hydrolases. As reported in the CAZy database (http://www.cazy.org/GH20.html), other GH20 members include, for example, β-hexosaminidases (EC 3.2.1.52) and lacto-*N*-bio­sidases (EC 3.2.1.140). Human HexA (a heterodimer of α and β subunits; Lemieux *et al.*, 2006 ▸), human HexB (a homodimer of β subunits; Maier *et al.*, 2003 ▸) and human *O*-GlcNAcase (Li *et al.*, 2017 ▸) are the best-characterized human enzymes, while *Sm*Chb from *Serratia marcescens* (Tews *et al.*, 1996 ▸), StrH from *Streptococcus pneumoniae* R6 (Jiang *et al.*, 2011 ▸) and *Sp*Hex from *Streptomyces plicatus* (Mark *et al.*, 2001 ▸) are bacterial GH20 members with well known functions and structures. Eukaryotic and bacterial GH20 members have different physiological functions and act on different substrates. Human HexA and HexB are key enzymes in glycolipid degradation (Federico *et al.*, 1991 ▸). Both are responsible for the hydrolysis of the terminal GalNAc residue from the GM2 ganglioside {GalNAcβ(1–4)-[NANAα(2–3)-]-Galβ(1–4)-Glc-ceramide} within lysosomes (Sandhoff & Kolter, 1998 ▸). Mutations in HexB genes often result in Sandhoff disease, whereas mutations in HexA decrease the hydrolysis of GM2 gangliosides and are the main cause of Tay–Sachs disease (Myerowitz, 1997 ▸; Mark *et al.*, 2003 ▸). In contrast, bacterial GH20 enzymes are part of the chitin-degradation pathway (Suginta *et al.*, 2010 ▸; Suginta, Chumjan, Mahendaran, Janning *et al.*, 2013 ▸; Thi *et al.*, 2014 ▸) and mainly degrade β-1,4 glycosidic bonds in chitin oligomers, generating GlcNAc monomers that are further metabolized as the sole source of cellular energy. The first crystal structure of a bacterial GH20 to be reported was that of *Sm*Chb. The structural details showed that *Sm*Chb (PDB entry 1qba) is a monomeric enzyme containing four functional domains, with the central (β/α)_8_ TIM-barrel domain acting as the catalytic domain (Tews *et al.*, 1996 ▸).

*Vh*GlcNAcase from *V. campbellii* strain ATCC BAA-1116 has already been characterized by our group as an exo-acting enzyme that sequentially degrades a small (*i.e.* shorter than six sugar units) chitooligosaccharide chain from the nonreducing end, generating GlcNAc monomers as the final products (Suginta *et al.*, 2010 ▸; Meekrathok & Suginta, 2016 ▸; Sirimontree *et al.*, 2016 ▸). The preferred substrate of *Vh*GlcNAcase is chitotetraose (GlcNAc_4_). The enzyme can hydrolyze colloidal chitin, but its specific activity for the polymeric substrate is much lower than for chitin fragments (Suginta *et al.*, 2010 ▸). We previously identified the gene encoding *Vh*GlcNAcase in the chromosome of *V. campbellii* and cloned it into the pQE60 expression vector, which is designed to be expressed in an *Escherichia coli* M15 (pREP4) host. In this study, we report the first crystal structures of a GH20 exo-β-*N*-acetylglucos­aminidase from the marine species *V. campbellii* in the absence and the presence of the natural substrate (GlcNAc)_2_, which was cleaved into GlcNAc during crystal soaking, so that one GlcNAc was bound in the active site. We also solved the structure of the D437A mutant of the same protein. Detailed crystallographic analysis revealed the important features of the enzyme for sugar binding and substrate specificity, and the roles of the active-site residues were elucidated by steady-state kinetic studies.

## 2. Experimental procedure   

### 2.1. Mutant design and site-directed mutagenesis   

Several active-site mutants were generated by the polymerase chain reaction (PCR) technique using the QuikChange Site-Directed Mutagenesis Kit (Stratagene, La Jolla, California, USA) according to the manufacturer’s protocols. The full-length *nag*2 gene encoding *Vh*GlcNAcase (residues 1–642), with ten additional residues at the C-terminus (RSRS residues of the cloning site followed by the His_6_ tag), was cloned into the pQE-60 expression vector (Qiagen, Valencia, California, USA) as described in Suginta *et al.* (2010 ▸) and the recombinant plasmid harboring the *nag*2 gene was used as a DNA template for mutagenesis experiments. Table 1 ▸ provides a list of the mutagenic primers (BioDesign, Bangkok, Thailand and Bio Basic Canada, Ontario, Canada) with the mutated codons underlined. The correct mutations were confirmed by automated DNA sequencing (First BASE Laboratories, Seri Kembangan, Malaysia).

### 2.2. Protein expression and purification   

Single colonies of the *E. coli* M15 (pREP4) host cells (Qiagen) transformed with the pQE60/*GlcNAcase* construct were picked and grown overnight at 37°C on Luria–Bertani (LB) agar plates containing 100 µg ml^−1^ ampicillin (Amp) and 25 µg ml^−1^ kanamycin (Kan). This overnight culture was used to inoculate a fresh 1 l culture using a 1:100 dilution. The fresh culture was Terrific Broth (TB) medium supplemented with 100 µg ml^−1^ Amp and 25 µg ml^−1^ Kan and was grown at 37°C with shaking at 200 rev min^−1^ until an OD_600_ of 0.6 was reached. To induce expression of recombinant *Vh*GlcNAcase, isopropyl β-d-1-thiogalactopyranoside (IPTG) was added to the culture medium to a final concentration of 0.4 m*M*. After 16 h of incubation at 20°C, the cell pellet was collected by centrifugation and resuspended in 20 ml extraction buffer [20 m*M* Tris–HCl pH 8.0 containing 150 m*M* NaCl, 1 m*M* phenylmethylsulfonyl fluoride (PMSF), 5%(*v*/*v*) glycerol, 1 mg ml^−1^ lysozyme and 1 mg ml^−1^ DNase I] and then lysed on ice using a Sonopuls Ultrasonic homogenizer with a 6 mm diameter probe (50% duty cycle; amplitude setting 30%; total time 20 s; 6–8 repeats). Insoluble debris and unbroken cells were removed by centrifugation at 12 000*g* at 4°C for 1 h and the supernatant was immediately applied onto a polypropylene column packed with 5 ml TALON Superflow metal-affinity resin (Clontech, USA) operated under gravitational flow at 4°C. After sample application, unbound proteins were washed out with eight column volumes (CV) of equilibration buffer (20 m*M* Tris–HCl pH 8.0 containing 150 m*M* NaCl) followed by 7 CV of the same buffer supplemented with 5 m*M* imidazole.

*Vh*GlcNAcase was eluted by applying 3 × 10 ml of 150 m*M* imidazole in the equilibration buffer at pH 8.0. Fractions containing active *Vh*GlcNAcase were then pooled and concentrated using Vivaspin-20 ultrafiltration membrane concentrators (Vivascience, Hanover, Germany) and further applied onto a HiPrep 16/60 Sephacryl S-200 column connected to an ÄKTAprime system (Amersham Bioscience, Piscataway, New Jersey, USA). *Vh*GlcNAcase-containing fractions were pooled and concentrated, and the protein purity was confirmed by 12% SDS–PAGE. The final concentration of *Vh*GlcNAcase was determined from the absorbance at 280 nm using a molar extinction coefficient of 118 720 *M* ^−1^ cm^−1^ (Gill & von Hippel, 1989 ▸). The freshly prepared protein was aliquoted, flash-frozen in liquid nitrogen and stored at −80°C until use.

### 2.3. Investigation of the protein state by size-exclusion chromatography   

The molecular weight (MW) of wild-type *Vh*GlcNAcase was investigated using size-exclusion chromatography. A HiPrep 26/60 Sephacryl S-300 prepacked column connected to an ÄKTAprime system (GE Healthcare Biosciences, Bangkok, Thailand) was equilibrated with 20 m*M* Tris–HCl buffer pH 8.0 containing 150 m*M* NaCl and was operated at a flow rate of 2.0 ml min^−1^. The gel-phase distribution coefficient (*K*_av_) of the analyte between the stationary and mobile phases was calculated as
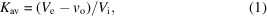
where *V*_e_ is the elution volume, *V*_o_ is the void volume and *V*_i_ is the volume of the stationary phase (Tayyab *et al.*, 1991 ▸). The column was calibrated with the protein standards ribonuclease A (13.7 kDa), ovalbumin (43 kDa), bovine serum albumin (BSA; 66 kDa), aldolase (158 kDa), ferritin (440 kDa) and thyro­globulin (669 kDa). Blue dextran 2000 was used to determine the void volume *V*_o_, while *N* ^ɛ^-DNP-l-lysine hydrochloride (0.35 kDa) was added as a control for the retention volume of each protein and also to determine the total volume of the column. A plot of the *K*_av_ of individual standard proteins (calculated from equation 1) versus the logarithm of the MW yielded a linear calibration plot, which allowed the MW of *Vh*GlcNAcase to be determined. To obtain the elution volume of *Vh*GlcNAcase, the purified enzyme (4 µg) mixed with *N* ^ɛ^-DNP-l-lysine was applied onto the HiPrep 26/60 Sephacryl S-300 gel-filtration column as specified. Fractions of 5 ml were collected, with *Vh*GlcNAcase being eluted in two peaks: the first eluted close to the void peak, while the second eluted near the BSA peak. SDS–PAGE analysis and GlcNAcase activity assays showed that the first peak was aggregated and inactive enzyme, while the second peak was the active enzyme. Therefore, the MW of the active *Vh*GlcNAcase was determined from the calibration curve as 76 kDa (expected value 74 kDa).

### 2.4. Protein crystallization, data collection and processing   

Preliminary crystallization of *Vh*GlcNAcase, both in the apo form and in complex with *N*-acetylglucosamine (GlcNAc), was performed as described elsewhere (Meekrathok *et al.*, 2015 ▸). Crystallization conditions were screened using sitting-drop vapor diffusion at 20°C with the commercially available screens The JCSG Core Suites I, II, III and IV, The Classics and Classics II Suites, The PACT Suite, The PEGs Suite and The Anions Suite (Qiagen, Hilden, Germany). Under the optimized crystallization condition, 1.5 µl wild-type *Vh*GlcNAcase solution (10 mg ml^−1^) was mixed with 1.5 µl reservoir solution consisting of 0.1 *M* sodium acetate pH 4.6, 1.4 *M* sodium malonate, while the D437A mutant was mixed with 0.1 *M* bis-Tris pH 7.5, 0.1 *M* sodium acetate, 20%(*w*/*v*) PEG 3350. 3D plate-shaped crystals of the wild type grew at 20°C within three days to dimensions of up to 400 × 200 × 20 µm. These wild-type crystals were subsequently successfully soaked with 10 m*M* (GlcNAc)_2_ in the corresponding mother liquor at 20°C for a period of approximately 30 min, as described previously (Meekrathok *et al.*, 2015 ▸). The native and soaked crystals were then transferred into a cryoprotectant solution containing the mother liquor with 2.9 *M* sodium malonate and 10 m*M* (GlcNAc)_2_. The D437A mutant grew in a condition consisting of 0.1 *M* bis-Tris pH 7.5, 0.1 *M* sodium acetate, 20%(*w*/*v*) PEG 3350 and was transferred into a cryoprotectant solution consisting of mother liquor supplemented with 25%(*v*/*v*) glycerol. X-ray diffraction data were collected from all crystals at 100 K on the PX-II beamline at the Swiss Light Source in Villigen, Switzerland using a PILATUS 6M detector. The data-collection strategy was determined with *iMosflm* (Battye *et al.*, 2011 ▸) from the *CCP*4 suite (Winn *et al.*, 2011 ▸) and the diffraction data were indexed, integrated and scaled using *XDS* (Kabsch, 2010 ▸). Crystallographic and refinement statistics are summarized in Table 2 ▸.

### 2.5. Structure determination and refinement   

Molecular replacement (MR) was employed to obtain phase information using *Phaser* (McCoy *et al.*, 2007 ▸) from the *CCP*4 suite with the structure of β-hexosaminidase from *Arthrobacter aurescens* (PDB entry 3rcn; 35% identical to GlcNAcase from *V. campbellii*; Midwest Center for Structural Genomics, unpublished work) as a search model. The final model of wild-type *Vh*GlcNAcase was subsequently used as a template to obtain the phases for the data sets for the *Vh*GlcNAcase–substrate complex and the D437A mutant. Model building was performed by iterative cycles consisting of manual building in *Coot* (Emsley *et al.*, 2010 ▸) and restrained refinement in *REFMAC*5 from the *CCP*4 suite (Winn *et al.*, 2011 ▸; Murshudov *et al.*, 2011 ▸). During the model-rebuilding process, electron density for only one GlcNAc molecule could be found in the structure even though the crystal was soaked with (GlcNAc)_2_. The molecular topology of GlcNAc was taken from the Protein Data Bank (PDB entry 3gh5) and then modeled into the corresponding 2*F*_o_ − *F*_c_ and *F*_o_ − *F*_c_ maps. The crystallographic data and refinement statistics of the finalized model of the *Vh*GlcNAcase structures are summarized in Table 2 ▸. The geometry of each final model was verified by *PROCHECK* (Laskowski *et al.*, 1993 ▸) and *MolProbity* (Chen *et al.*, 2010 ▸). Ligand–protein interactions were analyzed using *LigPlot*+ (Laskowski & Swindells, 2011 ▸), and the graphical structures and electron-density maps were visualized using *PyMOL* (DeLano, 2002 ▸).

### 2.6. GlcNAcase activity assay   

GlcNAcase activity was determined by a colorimetric assay using 4-nitrophenyl *N*-acetyl-β-d-glucosaminide (*p*NP-GlcNAc; Sigma–Aldrich, St Louis, Missouri, USA) as a substrate. The reaction of 0.1–5 µg protein samples with 125 µ*M* *p*NP-GlcNAc in 100 m*M* sodium phosphate buffer pH 7.0 in a total volume of 200 µl was carried out in triplicate in a 96-well microtiter plate at 37°C for 10 min with constant agitation in a ThermoMixer Comfort (Eppendorf AG, Hamburg, Germany). The reaction was terminated by the addition of 100 µl 3 *M* sodium carbonate. The amount of 4-nitrophenol (4-NP) released was monitored optically at a wavelength of 405 nm using a Benchmark Plus microplate spectrophotometer (Bio-Rad Laboratories, Hercules, California, USA). A calibration curve of a 4-NP standard varying from 0 to 20 nmol was constructed, allowing determination of the molar quantity of 4-NP liberated by the enzymatic reaction. The specific hydrolytic activity of the enzyme was expressed as nanomoles of 4-NP produced in 1 min at 37°C.

### 2.7. Steady-state kinetic measurements   

Kinetic studies of wild-type and mutant *Vh*GlcNAcase were carried out using a colorimetric assay in a microtiter plate reader, as described above, with substrate concentrations varying from 0 to 500 µ*M*. Briefly, a 200 µl reaction mixture consisting of 0–500 µ*M* *p*NP-GlcNAc in 100 m*M* sodium phosphate buffer pH 7.0 and 0.1–30 µg enzyme was incubated at 37°C with constant shaking for 10 min. The enzymatic reactions were then terminated by adding 100 µl 3 *M* sodium carbonate. The amount of reaction product was measured at 405 nm and converted to molar quantities using a calibration curve for 4-NP as described previously. The kinetic parameters (*K*_m_, *k*_cat_ and *k*_cat_/*K*_m_) were determined from triplicate assays using the Michaelis–Menten function in *GraphPad Prism* version 0.6.0 (GraphPad Software, San Diego, California, USA).

## 3. Results   

### 3.1. Crystallization, refinement statistics and crystal structures   

Wild-type *Vh*GlcNAcase and its catalytic mutant D437A (hereafter referred to as WT and D437A, respectively) were expressed at high levels in *E. coli* M15 (pREP4) cells as C-terminally His_6_-tagged polypeptides and were purified to homogeneity on a cobalt-affinity column (Clontech, USA) followed by gel-filtration chromatography, giving a final yield of approximately 15–20 mg purified enzyme per litre of bacterial culture. Single crystals of apo WT and apo D437A were successfully grown by hanging-drop vapor diffusion under the optimized conditions described in Section 2 and diffraction data were obtained using synchrotron X-ray radiation to resolutions of 2.37 and 2.6 Å, respectively. A single crystal of WT *Vh*GlcNAcase soaked with (GlcNAc)_2_ diffracted to a resolution of 2.50 Å (Table 2 ▸). All crystals belonged to space group *P*2_1_ with two molecules per asymmetric unit. The structures of all crystal forms were determined by the MR method and refined to *R*_work_ and *R*_free_ values of 0.21 and 0.25, respectively, for apo WT, 0.21 and 0.26, respectively, for WT GlcNAc and 0.21 and 0.24, respectively, for apo D437A. The root-mean-square deviations (r.m.s.d.s) of bond lengths and angles of all crystals were refined to between 0.007 and 0.009 and between 1.20 and 1.34, respectively. The average *B* factors refined to 55.34 Å^2^ for apo WT, 36.35 Å^2^ for WT GlcNAc and 64.96 Å^2^ for apo D437A. The coordinates and structure factors were deposited in the Protein Data Bank with PDB codes 6ezr for apo WT, 6ezs for the WT–GlcNAc complex and 6ezt for apo D437A.

**Figure 1:**
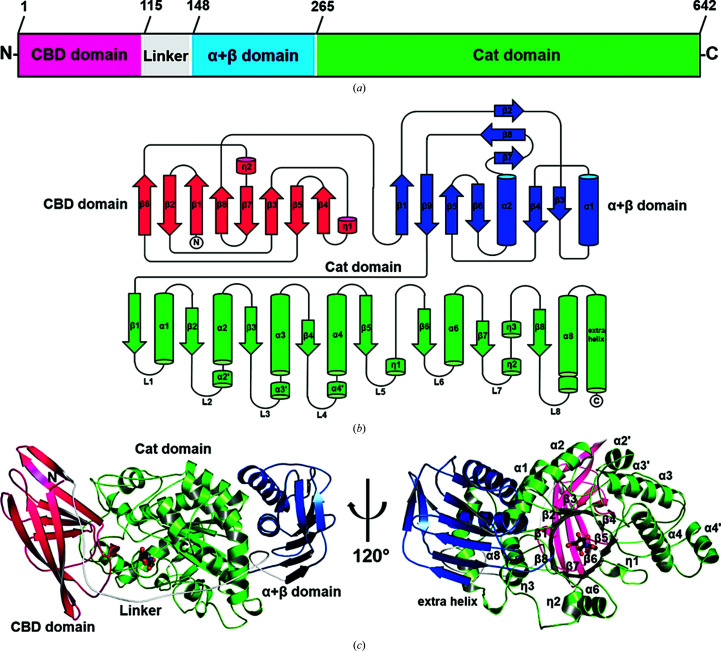
Domain organization and overall crystal structure of *Vh*GlcNAcase. (*a*) Domain composition of the *Vh*GlcNAcase sequence. (*b*) Topology diagram of *Vh*GlcNAcase analyzed with the *PDBSum* server. The carbohydrate-binding domain (residues 1–114) is represented in dark pink, the α+β domain (residues 148–259) in blue and the (β/α)_8_ TIM-barrel catalytic domain (residues 292–633) in green. Eight β-strands are labeled β1–β8 and the six regular α-helices connecting the β-strands are labeled α1–α8. Short helices are depicted by cylinders labeled η. Segmented secondary-structure elements are denoted α′, for instance α2′ indicates the short helix within the region of the α2 helix. (*c*) Ribbon representation of the overall structure of *Vh*GlcNAcase, consisting of three domains. The N-terminal carbohydrate-binding (CBD) domain is presented in dark pink, the α+β domain is in blue, a linker between the CBD domain and the α+β domain is in gray and the TIM-barrel catalytic (Cat) domain is in green. The GlcNAc molecule in the active site of *Vh*GlcNAcase is shown as a black ball-and-stick model with C atoms in black, N atoms in blue and O atoms in red.

Fig. 1 ▸(*a*) shows the domain arrangement of *Vh*GlcNAcases based on the 3D structure obtained from this study. The overall structure contains three distinct domains: an N-terminal carbohydrate-binding (CBD) domain (residues 1–114; pink) connected by a long linker to an α+β domain (residues 148–264; blue) followed by a C-terminal catalytic (Cat) domain (residues 265–642; green) (Val-Cid *et al.*, 2015 ▸). Fig. 1 ▸(*b*) is a topology diagram showing details of the secondary-structural elements of *Vh*GlcNAcase. The CBD domain consists primarily of eight antiparallel strands (β1–β8) that form an immunoglobulin-like fold, while the α+β domain is composed of two helices mixed with six strands flanked by three additional short strands that are aligned out of the main plane of this domain. The central Cat domain has a typical (β/α)_8_ TIM-barrel-like fold, in which the helices and strands alternate in an antiparallel fashion. Note that in the TIM-barrel domain the fifth helix (α5), which joins strands β5 and β6 of the canonical TIM-barrel fold, and the seventh helix (α7), which joins strands β7 and β8, are missing and are replaced by three short helical (η) segments. Helix η1 connects strands β5 and β6, and the η2 and η3 segments connect strands β7 and β8.

We also observe an additional long helix at the end of helix α8, which is also seen in *Sp*Hex (PDB entry 1m01; Williams *et al.*, 2002 ▸). Fig. 1 ▸(*c*) shows the structural architecture of *Vh*GlcNAcase, in which the central Cat domain is flanked by the CBD domain and the α+β domain. This CBD domain is structurally related to the family 2 carbohydrate-binding module (CBM2) in the CAZy database (Lombard *et al.*, 2014 ▸). A *DALI* search (http://ekhidna.biocenter.helsinki.fi; Holm & Sander, 1993 ▸) reveals that the CBD domain of *Vh*GlcNAcase has the closest structural identity to the CBD of endoglucanase D from *Clostridium cellulovorans* (*Z*-score = 13.1; r.m.s.d. of 1.8 Å over 96 residues; 13% sequence identity; PDB entry 3ndz; C. M. Bianchetti, R. W. Smith, C. A. Bingman & G. N. Phillips Jr, unpublished work). The α+β domain is similar to the α+β domain of β-Hex from *A. aurescens* (*Z*-score = 17.0; r.m.s.d. of 1.5 Å over 112 residues; 28% sequence identity; PDB entry 3rcn). The function of this domain is unknown, but it may help to solubilize and stabilize the catalytic domain (Val-Cid *et al.*, 2015 ▸). Lastly, the closest relative of the catalytic domain of *Vh*GlcNAcase is the TIM-barrel structure of β-Hex from *A. aurescens* (*Z*-score = 45.9; r.m.s.d. 1.7 Å over 347 residues; 39% sequence identity; PDB entry 3rcn).

In *Vh*GlcNAcase 12 cysteine residues are distributed over the three protein domains. The previously reported structure of β-hexosaminidase from *S. plicatus* (*Sp*Hex; PDB entry 1m01) suggested one disulfide bond in the Cat domain between Cys263 and Cys282 (Williams *et al.*, 2002 ▸), which is not present in *Vh*GlcNAcase. Although there are two neighboring cysteine residues very close to the active site in *Vh*GlcNAcase (Cys272 and Cys583), these residues are not conserved in *Sp*Hex and their side chains are too far apart (4.5 Å) to form a disulfide bridge.

### 3.2. Dimer interface   

**Figure 2:**
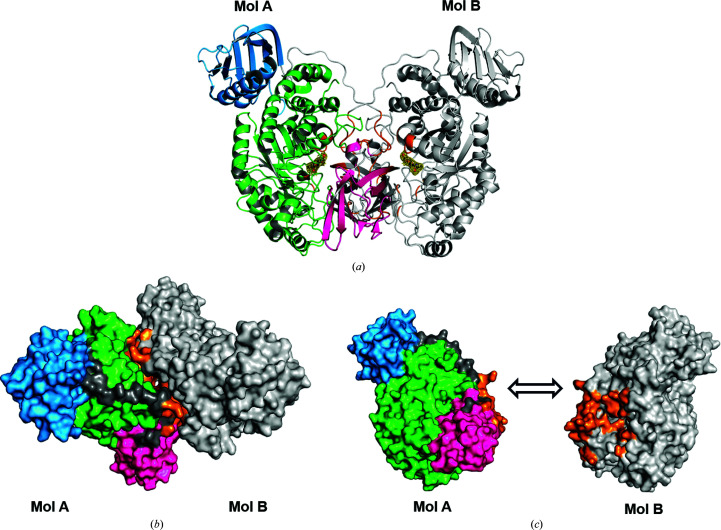
The dimer interface in the *Vh*GlcNAcase crystals. (*a*) The overall crystal structure of *Vh*GlcNAcase with two identical molecules per asymmetric unit. The GlcNAc product found in the active site of each molecule is shown in a black ball-and-stick representation, with the *F*_o_ − *F*_c_ electron density shown as a yellow mesh. (*b*) Surface representation of the dimer in the asymmetric unit. For Mol A, the Cat domain is shown is green, the α+β domain in cyan, the CBD domain in magenta and the linker that joins the Cat and CBD domains in gray. Mol B is represented in gray, while the dimer interface is colored orange. (*c*) A separate depiction of the dimer interface in the same orientation as in (*a*), with the dimer interface area highlighted in orange.

The crystallographic analysis showed that the single crystals of apo (PDB entry 6ezr) and holo (PDB entry 6ezs) *Vh*GlcNAcase (in complex with GlcNAc) contained two identical (r.m.s.d. of 0.14–0.16 Å over 639 C^α^ atoms) molecules per asymmetric unit (Meekrathok *et al.*, 2015 ▸). The two protein molecules, designated Mol A and Mol B, are related by a twofold rotational axis, as seen in Fig. 2 ▸(*a*). Each holoenzyme molecule contains one GlcNAc unit (*F*_o_ − *F*_c_ density shown as an orange mesh with the sugar shown as sticks) at subsite −1 of the catalytic pocket (the Cat domain of Mol A is in green and that of Mol B in gray). The entrance to the substrate-binding cleft of the Cat domain is covered by the CBD domain (magenta for Mol A versus gray for Mol B) of the neighboring molecule. Notably, the WT crystals were soaked with the substrate (GlcNAc)_2_ to obtain the ligand-bound structure, but we observed only a single GlcNAc in the active site of the enzyme. Given that (GlcNAc)_2_ is a stable sugar, it was presumed that (GlcNAc)_2_ was hydrolyzed during crystal soaking, leaving a GlcNAc product in the high-affinity site (site −1). Fig. 2 ▸(*b*) presents a surface representation of the molecular packing of Mol A and Mol B of WT *Vh*GlcNAcase in complex with GlcNAc, with the residues in the dimer interface highlighted in orange. Fig. 2 ▸(*c*) shows Mol A and Mol B separately in the same orientation as in Fig. 2 ▸(*a*). The interface region (orange) covers 4054.87 Å^2^ of the total protein surface area (66 973.06 Å^2^). 19 residues (Val12, Leu13, Ser14, Glu15, Gln16, Lys17, Gln18, Asn19, Arg21, Asp44, Arg45, Asp50, Ser51, Val52, Ser53, Ser87, Asn88, Pro89 and Arg91) from the CBD domain, 12 residues (Ile397, Glu438, Asn441, Glu489, Trp505, Leu506, Ser507, Glu509, Gln527, Trp546, Ala547 and Asn548) from the Cat domain and six residues (Val121, Ala123, Ser124, Pro125, Tyr126 and Arg127) from the linker are involved in the dimer interface; all are mostly hydrophilic, consistent with the finding that *Vh*GlcNAcase is a monomer in solution, as shown below.

### 3.3. Molecular-weight determination   

**Figure 3:**
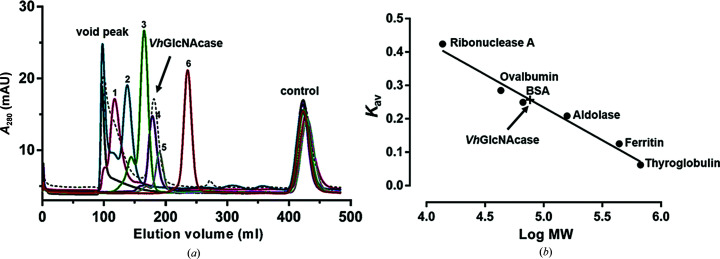
Size-exclusion chromatographic profile and calibration curve of *Vh*GlcNAcase and standard proteins. (*a*) The HiPrep 26/60 Sephacryl S-300 prepacked column was calibrated with six well defined globular protein standards plus the small molecule *N* ^ɛ^-DNP-l-lysine, ranging from 0.35 to 669 kDa. *N*_ɛ_-DNP-l-lysine (0.35 kDa) was used to estimate the internal volume of the column and blue dextran 2000 was used to determine the void fraction. (*b*) The estimated molecular mass of *Vh*GlcNAcase (76 kDa, expected 74 kDa) was determined from the calibration plot of *K*_av_ versus log MW after the *K*_av_ value had been calculated from the measured elution volume.

*Vh*GlcNAcase was previously suggested to be a monomeric enzyme by native PAGE analysis (Meekrathok *et al.*, 2015 ▸), but in the crystal structure two molecules per asymmetric unit were observed. To clarify this point, we carried out size-exclusion chromatography (SEC) to determine the apparent MW of *Vh*GlcNAcase in its native form. The chromatographic profiles of *Vh*GlcNAcase and six calibration proteins are shown in Fig. 3 ▸(*a*). The retention volume of 181 ml for *Vh*GlcNAcase in the chromatographic profile was converted to the distribution coefficient (*K*_av_), which corresponds to a MW of 76 kDa on the calibration curve plotted for *K*_av_ and log_10_ MW (Fig. 3 ▸ *b*). Compared with the expected MW of *Vh*GlcNAcase (74 kDa), the MW obtained from gel filtration clearly confirms that *Vh*GlcNAcase is a monomer in solution. Thus, the dimer observed in the crystals is likely to be a crystallization artifact induced by the particularly high protein concentration needed for crystal formation. We also carried out a *PISA* analysis (Krissinel & Henrick, 2007 ▸) and the results predicted no stable dimer formation in solution, confirming the SEC results.

### 3.4. Interactions with the sugar in the active site   

The complex of WT *Vh*GlcNAcase with substrate shows one molecule of GlcNAc occupying the −1 subsite (subsites were assigned based on the structure of *Sm*Chb in complex with chitobiose; Tews *et al.*, 1996 ▸). The single GlcNAc was most likely to be produced by cleavage of the soaked substrate (GlcNAc)_2_ by active WT enzyme on the surface of the crystal during the soaking time of approximately 30 min. The long loop that connects the β1–β2 hairpin of Mol B close to the entrance to the active site and Gln16 would clearly obstruct a long chitooligosaccharide chain from entering the active site and may explain why we did not observe any ligand molecule in the active site of the catalytically inactive D437A mutant co-crystallized with (GlcNAc)_2_.

**Figure 4:**
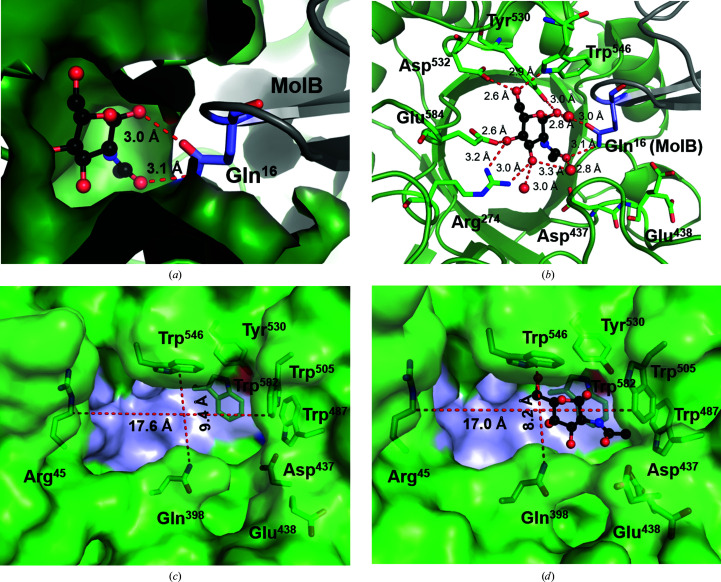
Analysis of sugar–enzyme interactions. (*a*) Cartoon representation, focusing on Gln16 of Mol B that forms two strong hydrogen bonds to −1 GlcNAc, partially obstructing the entrance to the active site of Mol A. (*b*) Hydrogen bonds are formed between the bound GlcNAc (black sticks) and the sugar-binding residues (green/blue sticks) around subsite −1. The interactions were generated at 3.5 Å distance by *PyMOL*. The C atoms of the binding residues are shown in green and the sugar molecules in black, with N atoms in blue and O atoms in red. Water molecules that help to mediate the interactions are presented as red balls. (*c*) Surface representation of the sugar-binding pocket of the unliganded form of *Vh*GlcNAcase. (*d*) Surface representation of the sugar-binding pocket of the *Vh*GlcNAcase–GlcNAc complex, showing local changes causing closure of the substrate-binding pocket around subsite −1 relative to the binding pocket in apo *Vh*GlcNAcase.

In the holo structure, the glycosidic O atom (O_1_) at the anomeric C atom of the −1 GlcNAc ring makes a hydrogen bond to the amide side chain of Gln16 on the loop belonging to Mol B. The side chain of Gln16 of Mol B also makes a second hydrogen bond to the O_7_ atom of the C_2_ acetamido group of −1 GlcNAc (Fig. 4 ▸ *a*). The −1 GlcNAc adopted a ^4^ *C*_1_ chair conformation based on the Cremer–Pople parameter calculator (Cremer & Pople, 1975 ▸; Jeffrey & Yates, 1979 ▸). As shown in Fig. 4 ▸(*b*), the reducing end (C_1_ OH) of −1 GlcNAc makes two hydrogen bonds to the side chain of Gln16 of Mol B and Tyr530, and the C_2_ acetamido group is immobilized by the side chain of Gln16 of Mol B and a water molecule. The C_3_ OH forms two hydrogen bonds to nearby water molecules and a hydrogen bond to the side chain of Arg274. The nonreducing end (C_4_ OH) is immobilized by the side chains of Arg274 and Glu584, respectively. The C_6_ OH forms two hydrogen bonds to the side chains of Asp532 and the N atom of the Trp546 side chain. The sugar–enzyme interactions shown in Fig. 4 ▸(*b*) are within a distance of 3.5 Å.

The predicted catalytic pair Asp437–Glu438 is located near the C_2_ acetamido group of the bound sugar. Asp437 makes a hydrogen bond to the carbonyl residue of the C2 moiety, while the side chain of Glu438 was seen in two conformations, each with 0.5 occupancy. The first rotamer is pointing away, while the second rotamer points towards the sugar molecule. The side chain of Gln16 of Mol B interferes with its ideal location close to the glycosidic O atom. Fig. 4 ▸(*c*) shows a surface representation of the empty subsite −1 of apo WT *Vh*GlcNAcase (PDB entry 6ezr) that is surrounded by aromatic residues. Four conserved aromatic residues, Trp487, Trp505, Tyr530, Trp546 and Trp582, essentially create the pocket wall. Superimposition of Mol A of the apo *Vh*GlcNAcase structure onto Mol A of the structure in complex with GlcNAc gives an r.m.s.d. of 0.90 Å over 1278 residues (two chains) and an r.m.s.d. of 0.26 Å^2^ over 639 residues, *i.e.* there are no significant differences between the CBD domain and the α+β domain of the two enzyme forms. When compared with the catalytic pocket of the unliganded enzyme (Fig. 4 ▸ *c*), a small movement of the loop regions surroundings subsite −1 of the enzyme can be observed. The dimensions of the GlcNAc-fitted catalytic cleft (8.2 × 17.0 Å^2^) are slightly narrower than the empty pocket (9.4 × 17.6 Å^2^) (Fig. 4 ▸ *d*). Four key side chains, Gln398 (part of loop L3), Asp437 and Glu438 (part of loop L4), and Trp505 (part of loop L6), are positioned close to the center of the catalytic pocket and are at an optimal distance to interact with the −1 GlcNAc.

### 3.5. Structural comparison of *Vh*GlcNAcase with other GH20 members   

**Figure 5:**
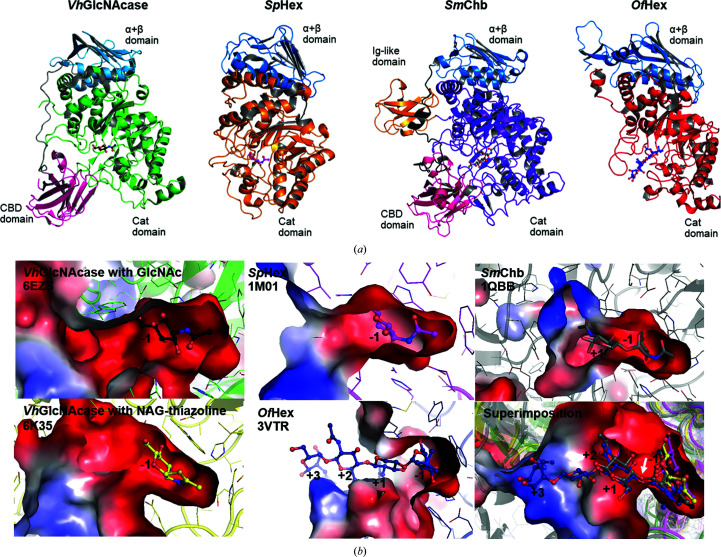
Structural comparison of *Vh*GlcNAcase with other bacterial GH20 structures in cartoon representation. (*a*) The structural domains of four related GH20 enzymes: *Vh*GlcNAcase in complex with GlcNAc (PDB entry 6ezs), *Sp*Hex in complex with GlcNAc (PDB entry 1m01), *Sm*Chb in complex with (GlcNAc)_2_ (PDB entry 1qbb) and *Of*Hex in complex with TMG-chitotriomycin (PDB entry 3vtr), are shown in the same orientation. (*b*) The molecular surfaces around the active sites are colored by the electrostatic potential calculated from the crystal structures with *PyMOL*. The active sites of all four structures are very acidic (red surface) and form more or less deep clefts to accommodate the oligosaccharides. Each structure is in the same orientation and the substrates of *Vh*GlcNAcase (GlcNAc or NAG-thiazoline), *Sp*Hex (GlcNAc) and *Of*Hex (TMG-chitotriomyocin) are superimposed with (GlcNAc)_2_ from the *Sm*Chb structure in the bottom right picture.

**Figure 6:**
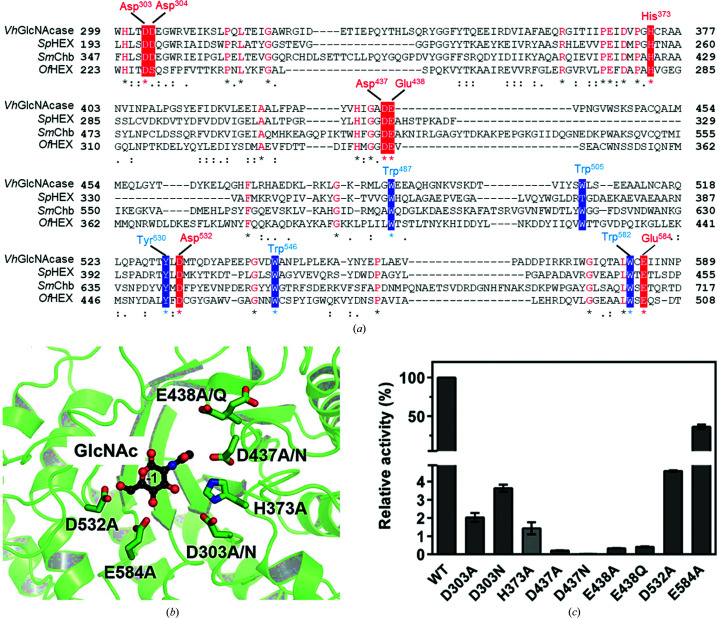
Active-site mutational design. (*a*) Sequence alignment of the catalytic domains of four GH20 enzymes: *Vh*GlcNAcase, *Sp*Hex, *Sm*Chb and *Of*Hex. Their amino-acid sequences were retrieved from the PDB using the PDB codes presented in Fig. 5 ▸. The sequence alignment was carried out by *Clustal Omega* (https://www.ebi.ac.uk/Tools/msa/clustalo/) and displayed by *Jalview* version 2.11.1.3 (https://www.jalview.org/). The conserved polar residues equivalent to Asp303, Asp304, His373, Asp437, Asp438 and Glu584 of *Vh*GlcNAcase are shaded red, while the conserved aromatic residues equivalent to Trp487, Trp505, Tyr530, Trp546 and Trp582 are shaded blue. (*b*) The positions of the mutated residues Asp303, His373, Asp437, Asp438 and Glu584 in the catalytic pocket surrounding the −1 GlcNAc molecule. Colors: green for C atoms of the binding residues and black for the C atoms of the sugar molecule, blue for N atoms and red for O atoms. (*c*) Bar graphs representing the relative enzymatic activity of the active-site mutants in *p*NP-GlcNAc hydrolysis in comparison of that of WT *Vh*GlcNAcase.

The crystal structure of *Vh*GlcNAcase in complex with GlcNAc is similar to those of other GH20 β-*N*-acetylhexos­aminidases, including the WT β-hexosaminidase *Sp*Hex from *S. plicatus* (PDB entry 1m01; r.m.s.d. of 2.1 Å over 445 residues; Mark *et al.*, 2001 ▸), the chitobiase *Sm*Chb from *S. marcescens* (PDB entry 1qbb; r.m.s.d. of 2.5 Å over 613 residues; Tews *et al.*, 1996 ▸) and the insect β-*N*-acetyl-d-hexos­aminidase *Of*Hex1 E328A (PDB entry 3vtr, r.m.s.d. of 2.7 Å over 456 residues; Liu *et al.*, 2011 ▸). Fig. 5 ▸(*a*) shows the domain structures of the four GH20 GlcNAcase orthologs. As mentioned earlier, *Vh*GlcNAcase contains three distinct domains, an N-terminal CDB domain (magenta), an α+β domain (cyan) and a C-terminal Cat domain (green), while *Sp*Hex contains only two domains: an α+β domain (cyan) connected to a C-terminal Cat domain (brown). The N-terminal CBD domain is missing in *Sp*Hex. *Sm*Chb consists of four domains designated as an N-terminal CBD domain (magenta) followed by an α+β domain (cyan), a TIM-barrel Cat domain (lilac) and a C-terminal immunoglobulin (IgG)-like domain (orange). The structure of insect *Of*Hex is similar to that of *Sp*Hex, and contains two domains: an α+β domain (cyan) connected to a C-terminal Cat domain (red). Note that the Cat domain of the four enzymes has a common (β/α)_8_-barrel fold and is conserved among all GH20 GlcNAcases. Fig. 5 ▸(*b*) shows the electrostatic surface around the active sites of *Vh*GlcNAcase complexed with GlcNAc (PDB entry 6ezs) and NAG-thiazoline (PDB entry 6k35; Meekrathok *et al.*, 2020 ▸), *Sp*Hex with GlcNAc (PDB entry 1m01), *Sm*Chb with (GlcNAc)_2_ (PDB entry 1qbb) and *Of*Hex1 E328A in complex with TMG-chitotriomycin (PDB entry 3vtr). The sugar-binding pockets of these GH20 GlcNAcases are highly polar due to six conserved proton-donating groups that form a strongly negatively charged surface (red) around subsite −1 of the active site of each enzyme [see also Fig. 6 ▸(*a*) for the positions of these residues in the corresponding amino-acid sequences]. Further comparison of the substrate-binding pockets and the positions of the bound ligands of the above-mentioned GH20 enzymes suggests that the active sites of *Vh*GlcNAcase and *Of*Hex have elongated pockets with an open end towards the positive subsites (subsites +1, +2 and +3), allowing the accommodation of a chitooligosaccharide chain of 2–4 units. The active sites of *Sp*Hex and *Sm*Chb, on the other hand, are small and rather short open pockets that are suitable for accommodating only one or two GlcNAc units. The sugar ligand of at least one unit is found at subsite −1, which is located at the closed end of the tunnel/pocket. In all enzymes, cleavage occurs between subsites −1 and +1 [indicated by a white arrow, Fig. 5(*b* ▸)]. The summary of ligand–active-site residue interactions for all of the examined GH20 enzymes is presented in Table 3 ▸. *LIGPLOT* analysis showed that both hydrogen bonds and hydrophobic interactions between the sugar ligand and the active-site residues of each enzyme are mainly formed at the most favored site −1. Many fewer interactions are seen at the product site +1 even with two units of sugar, such as chitobiose in *Sm*Chb or TMG-chitomyosin in *Of*HEX.

### 3.6. Identification of the active-site residues directly involved in catalysis by *Vh*GlcNAcase   

We have previously proposed that the catalytic mechanism for the cleavage of the glycosidic bond requires a catalytic pair located near the cleavage site in the sugar chain (Suginta *et al.*, 2010 ▸; Meekrathok & Suginta, 2016 ▸). Since we only observe the GlcNAc cleavage product at subsite −1, and the glycosidic O atom at the anomeric C atom of the bound sugar is in direct contact with Gln16 of Mol B instead of the catalytic residue Glu438 due to a crystal-packing artifact, we cannot directly observe the catalytically relevant conformation. Fig. 6 ▸(*a*) shows an amino-acid alignment of three sequence segments of the catalytic domains of the enzymes shown in Fig. 5 ▸.

*Vh*GlcNAcase has 29% identity overall to *Sp*Hex, 28% identity to *Sm*Chb and 24% identity to *Of*Hex. The positions of the conserved active-site residues equivalent to Asp303, Asp304, His373, Asp437, Glu438, Asp532 and Glu584 of *Vh*GlcNAcase are highlighted in red. Note that Tyr530, Trp546 and Trp582 (residues highlighted in blue), which make hydrophobic contacts with the −1 GlcNAc, are also conserved among the four GH20 enzymes. Obviously, the acidic pair (Asp437–Glu438 in *Vh*GlcNAcase) is completely conserved and was also identified as the catalytic pair in *Sp*Hex (Asp313–Glu314; Mark *et al.*, 2001 ▸), *Sm*Chb (Asp539–Glu540; Tews *et al.*, 1996 ▸) and *Of*Hex (Asp327–Glu328) (Liu *et al.*, 2011 ▸). Fig. 6 ▸(*b*) shows the positions of these amino-acid side chains around the −1 GlcNAc molecule in *Vh*GlcNAcase. To elucidate the impact of these residues on catalysis, they were chosen as targets for kinetic assessment. Site-directed mutagenesis generated eight single mutants, designated D303A, D303N, D437A, D437N, E438A, E438Q, H373A, D532A and E584A. All of these mutants showed drastic decreases in the enzyme activity, as measured by hydrolysis of *p*NP-GlcNAc, especially the mutants of the catalytic residues D437A/N and E438A/Q (Fig. 6 ▸ *c*), confirming the critical role of the conserved Asp437–Glu438 pair in substrate hydrolysis.

The Michaelis–Menten parameters of the hydrolytic activity of the *Vh*GlcNAcase variants were further analyzed and the values are presented in Table 4 ▸.

All of the mutants have significantly increased *K*_m_ and decreased *k*_cat_ values, yielding an overall decrease in the corresponding catalytic efficiency *k*_cat_/*K*_m_, relative to the values for the WT enzyme. As expected, the most severe loss in catalytic efficiency is observed with the catalytic mutant D437N, followed by D437A, E438A and E438Q. The D303A/N, H373A and D532A mutants show a moderate decrease in catalytic efficiency, while the E584A mutant showed a modest decrease in *k*_cat_/*K*_m_, which was only threefold less than that of the WT enzyme, suggesting that this residue does not play a critical role in catalysis.

### 3.7. Structural implications for substrate specificity   

**Figure 7:**
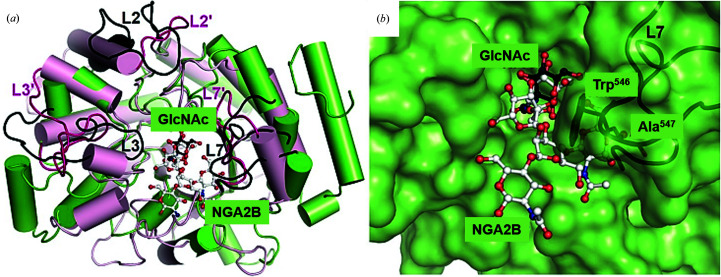
Comparison of substrate specificity based on the 3D structures of *Vh*GlcNAcase and exo-β-*N*-acetylglucosaminidase from *S. pneumoniae* (StrH). (*a*) Ribbon representation of *Vh*GlcNAcase (green and black) superimposed with StrH (PDB entry 2yla; pale pink and dark pink), showing the different orientations of the three long loops depicted as L2, L3 and L7 for *Vh*GlcNAcase and L2′, L3′ and L7′ for StrH. The NAG in the active site of *Vh*GlcNAcase is represented by a black ball-and-stick model, whereas the NGA2B in the active site of StrH is represented by a white ball-and-stick model. (*b*) Superposition of *Vh*GlcNAcase (green surface) complexed with GlcNAc (black ball-and-stick model) and StrH complexed with NGA2B (white ball-and-stick model), showing a putative steric clash of NGA2B due to Trp546 and Ala547 on loop 7 (L7) if it were bound to *Vh*GlcNAcase. The position of loop L7 (black loops in *Vh*GlcNAcase) close to the active site narrows the substrate-binding pocket in *Vh*GlcNAcase.

Exo-β-*N*-acetylglucosaminidase (StrH) from the pathogen *S. pneumoniae* is a GH20 homologue of *Vh*GlcNAcase, despite their relatively low sequence identity (17%). StrH is considered to be a virulence factor, recognizing complex GlcNAc-containing glycans as specific ligands (Jiang *et al.*, 2011 ▸). Superimposition of the catalytic domains of the two enzymes (PDB entry 6ezs for *Vh*GlcNAcase versus PDB entry 2yla for StrH) yielded a moderate r.m.s.d. of 2.2 Å over 265 residues (Jiang *et al.*, 2011 ▸). As shown in Fig. 7 ▸(*a*), the catalytic domains of the two enzymes are relatively dissimilar, which may contribute to their different substrate specificities. StrH is involved in the complete degradation of N-linked glycans of the human host (King, 2010 ▸), with degradation of the extracellular matrix component facilitating invasion of the host tissue by the pathogenic bacterium. The crystal structure of *Vh*GlcNAcase was compared with that of StrH in complex with the heteroglycan NGA2B {*N*-GlcNAc β-1,2-d-Man α-1,3 [*N*-GlcNAc β-1,2-d-Man α-1,6(*N*-GlcNAc β-1,4)]-d-Man β-1,4-*N*-GlcNAc} to investigate the substrate specificity of the enzyme. As shown in Fig. 7 ▸(*a*), the long loops of *Vh*GlcNAcase (in green and black) denoted L2 (β2–α2; residues 315–343), L3 (β3–α3; residues 386–406) and L7 (β7–β8; residues 525–551) significantly differ from those of StrH (in pale pink and dark pink), which has corresponding loops L2′ (residues 706–712), L3′ (residues 754–774) and L7′ (residues 898–914), respectively. Loop L3 has the highest thermal motion, with a *B* factor of around 40.6 Å^2^, followed by L2 and L7, which have *B* factors of approximately 34.3 and 26.9 Å^2^, respectively.

The long loops L2, L3 and L7 of *Vh*GlcNAcase (in black) protrude into the region where the NGAB2 substrate of StrH would be, which causes the substrate-binding region of *Vh*GlcNAcase to be narrower. Fig. 7 ▸(*b*) emphasizes a severe clash between the side chains of Trp546 and Ala547 of loop L7 in *Vh*GlcNAcase with the modeled NGA2B, suggesting that the active site of *Vh*GlcNAcase cannot possibly accommodate a branched oligosaccharide.

## 4. Discussion   

We successfully solved the crystal structures of three *Vh*GlcNAcase variants: two apo structures, of the wild type and the D437A mutant, and one holo structure, of the wild type in complex with (GlcNAc)_2_. After soaking the apo wild-type *Vh*GlcNAcase crystals with (GlcNAc)_2_, we observed a clear *F*_o_ − *F*_c_ density map corresponding to only one GlcNAc molecule in the −1 subsite. This GlcNAc is most likely to be the product of hydrolysis of (GlcNAc)_2_ by active WT enzyme on the surface of the crystal during the soaking time of 30 min. Soaking or co-crystallizing the D437A mutant with (GlcNAc)_2_ was not successful, *i.e.* no substrate was seen in the active site, probably owing to the entrance of the active site of one monomer in the asymmetric unit (Mol A) being blocked by the side chain of Gln16 of the last strand of the CBD domain of the second monomer (Mol B). Since the active site of *Vh*GlcNAcase is almost completely blocked by the neighboring monomer in the crystal structure, we assume that this would render such a dimeric enzyme extremely inefficient. This suggests that the active form of VhGlcNAcase is a monomer in solution, which could be confirmed by a gel filtration experiment. In addition, another crystal structure of *Vh*GlcNAcase in complex with NAG-thiazoline (NGT) shows a different space group (PDB entry 6k35; Meekrathok *et al.*, 2020 ▸) with an accessible active site and a completely different dimer in the asymmetric unit. This corresponds to the findings discussed in the review by Val-Cid *et al.* (2015 ▸), in which the bacterial GH20 enzymes most closely related to *Vh*GlcNAcase are described as being functional as monomers. Furthermore, a mutation of Gln16 would most likely not completely abolish the artificial dimerization since although the tip of the hairpin, including Lys17 and Gln18, blocks the entry to the ligand-binding pocket, the tightest interactions of the artificial dimer are outside the hairpin interaction site. The hairpin residues themselves interact relatively loosely. Lastly, we observed that monomeric *Vh*GlcNAcase in solution is fully active towards both natural chitooligosaccharide and artificial *p*NP-glycoside substrates (Suginta *et al.*, 2010 ▸). Such results indeed indicate that the dimeric interface does not interfere with the accessibility of substrates to the active site of the enzyme or with the catalytic activity of the enzyme.

The overall structures of apo WT and D437A mutant *Vh*GlcNAcases are essentially identical, both containing three distinct domains. The N-terminal CBD domain is followed by a relatively long linker that connects to the α+β domain. The α+β domain is similar to the GH20b domain in other GH20 β-hexosaminidases (Val-Cid *et al.*, 2015 ▸), but its function is still unknown. Some of its residues interact with the surface of the Cat domain and may help to stabilize the structural integrity of this domain. The largest domain is the catalytic (Cat) domain, which consists of eight strands alternating with six helices, instead of eight helices as reported in the related *N*-acetyl­glucosaminidases *Sm*Chb (PDB entry 1qbb; Tews *et al.*, 1996 ▸) and *Sp*Hex (DB entry 1m01; Mark *et al.*, 2001 ▸). The lack of helices α5 and α7 of the (β/α)_8_-barrel structure in the Cat domain and an additional helix at the end of helix α8 of *Vh*GlcNAcase seem to be common structural features of GH20 glycoside hydrolases (Maier *et al.*, 2003 ▸; Tews *et al.*, 1996 ▸; Mark *et al.*, 2001 ▸). In the closely related human HexB, the α-helices at positions α5 and α7 of the (β/α)_8_-barrel structure of the catalytic domain are also missing, and an additional C-terminal helix follows helix α8 (Maier *et al.*, 2003 ▸).

The active site of *Vh*GlcNAcases is strongly negatively charged, and analysis of the residues surrounding subsite −1 of the holo *Vh*GlcNAcase structure reveals that five acidic residues, Asp303, Asp437, Glu438, Asp532 and Glu584, around the surface of the active site, together with His373, are completely conserved among the four GH20 GlcNAcases. Although some of them are not directly in contact with the sugar molecule, these residues apparently contribute to the anionic character of subsite −1. Comparison of the active-site architecture and ligand–protein interactions among the four GH20 orthologs (Figs. 5 ▸ *a* and 5 ▸ *b*) provides some ideas about substrate specificity and subsite preference. Essentially, the catalytic pocket of *Vh*GlcNAcase is narrow and elongated, fitting a linear chitooligosaccharide chain of 2–4 GlcNAc units. The structure of the active site supports our previous quantitative HPLC and kinetic modeling of the enzymic reaction (Suginta *et al.*, 2010 ▸), which predicted the active site of *Vh*GlcNAcase to contain an array of most probably four binding subsites (−1, +1, +2 and +3). Based on this subsite topology, the cleavage site is located between subsites −1 and +1. In a recent study, we examined the inhibitory effect of NAG-thiazoline (NGT), a reaction-intermediate analog and a common inhibitor of GH20 GlcNAcases, and found that NGT strongly inhibited the hydrolytic activity of *Vh*GlcNAcase, with a *K*_i_ of 62 ± 3 µ*M* and a *K*_d_ of 32 ± 1.2 µ*M* (Meekrathok *et al.*, 2020 ▸). The structure of *Vh*GlcNAcase in complex with NGT showed that NGT occupies subsite −1 (Fig. 5 ▸ *b*, bottom left; PDB entry 6k35; Meekrathok *et al.*, 2020 ▸). The N atom at the C2 position of the bicyclic NGT mimics the N atom in the cyclized C2 acetamido group of the oxazolinium intermediate and makes a strong hydrogen bond to Asp437, while Glu438 is oriented in the optimal position for catalysis. These two residues are completely aligned with the catalytic pairs of β-Hex1 (PDB entry 3sur; Sumida *et al.*, 2011 ▸) and of *Sp*Hex (PDB entry 1hp5; Williams *et al.*, 2002 ▸; Vocadlo & Withers, 2005 ▸), both of which employ the substrate-assisted mechanism for catalysis. Although further kinetic experiments are required to prove the mode of action of *Vh*GlcNAcase, we assume that the enzyme, like other functionally characterized bacterial GH20 GlcNAcases, also employs this catalytic mechanism. In the substrate-assisted mechanism of glucosaminidases, two completely conserved acidic residues are proposed to be the catalytic pair (Meekrathok & Suginta, 2016 ▸; Jiang *et al.*, 2011 ▸; Mark *et al.*, 2001 ▸; Thi *et al.*, 2014 ▸). In *Vh*GlcNAcase, this acidic pair is identified as Asp437–Glu438, located next to the cleavage site. Given that *Vh*GlcNAcase is an exolytic enzyme that sequentially degrades a chitooligo­saccharide chain, releasing one GlcNAc at a time from the nonreducing end, the −1 GlcNAc would be identified as the remaining product after the hydrolysis of (GlcNAc)_2_ during crystallization. In both the apo and holo structures we observed an obstruction of the entrance of the active site by Gln16 from a neighboring molecule in the crystal. As shown by the SEC experiments, this is a crystallization artifact that would not occur in the monomeric enzyme in solution. The holo structure of *Vh*GlcNAcase with GlcNAc provides a snapshot of the sugar ring in the ^4^ *C*_1_ conformation, which signifies the most favorable form of the sugar. The same sugar conformation was also observed in the active site of the β-hexosaminidase Hex1T from *Paenibacillus* sp. TS12 (PDB entry 3gh5; Sumida *et al.*, 2009 ▸) and *Sp*Hex from *S. plicatus* (PDB entry 1m01; Mark *et al.*, 2001 ▸).

Structural comparison of the apo and holo forms of *Vh*GlcNAcase provides evidence of a ligand-induced conformational change in the local area of the active site. The most notable effect is caused by a swinging of the side chain of Glu438 in the holo structure towards the sugar ring, causing some movement of the corresponding loop L4, essentially narrowing the catalytic cavity around the −1 subsite by 0.6 Å (in length) × 1.2 Å (in width), while other parts of the active site remain unaltered. This induced-fit movement of Glu438 enables the bond cleavage of chitooligosacharide bound between subsites −1 and +1, while the positioning of Asp437 may help the enzyme to stabilize the transition state by interaction with the acetamido group of the substrate through a substrate-assisted catalysis mechanism (Mark *et al.*, 2001 ▸). Point mutation of Asp437 to Ala/Asn and Glu438 to Ala/Gln generated mutants with almost no GlcNAcase activity, confirming the catalytic functions of these two residues. The reduction in catalytic activity of the D437A and D437N mutants is due to the loss of the negative charge of the side chain of this amino acid, which results in destabilization of the reaction intermediate in the transition state (Meekrathok & Suginta, 2016 ▸; Mark *et al.*, 2001 ▸). Mutation of Glu438 to Ala/Gln instead removes the proton-donating group that is mandatory for bond cleavage in acid catalysis. A complete loss of catalytic activity was previously seen on changing the equivalent acidic pairs in *Sp*Hex (Asp313–Glu314; Mark *et al.*, 2001 ▸), *Sm*Chb (Asp539–Glu540; Tews *et al.*, 1996 ▸) and *Of*Hex1 (Asp327–Glu328; Liu *et al.*, 2011 ▸). Although they are all members of the GH20 family, *Vh*GlcNAcase has only <30% sequence identity to the other three GH20 enzymes (24% to *Of*HEX and 29% to *Sm*Chb and SpHEX), and the dissimilarities in the shape and the surface-charge properties of the sugar-binding pocket from those of the other enzymes highlights some novel features of this enzyme. Its elongated and shallow catalytic pocket is suited to accommodating a linear chitooligosaccharide chain of 2–4 units (Suginta *et al.*, 2010 ▸). The highly anionic surface along subsites +1, −1, −2 and −3 supports the formation of a large network of hydrogen-bond interactions between the binding residues and the corresponding sugar moieties, which essentially determine the strong binding affinity of *Vh*GlcNAcase for its substrate. In contrast, the catalytic pockets of *Sm*Chb and *Sp*HEX are shorter but wider, suggesting that these two enzymes act preferentially on small substrates, *i.e.* sugar dimers. The catalytic cleft of *Of*HEX is more open and less homogenous in the charged surface, signifying a broad substrate specificity of this enzyme towards hetero GlcNAc-containing molecules. Although the catalytic domains of GH20 share a highly conserved (β/α)_8_ TIM-barrel architecture, some GH20 enzymes, such as StrH, HexA and HexB, recognize a broad range of sugar substrates, including branched β(1–4), β(1–3), β(1–2) and β(1–6) glycosidic-linked GlcNAc-containing glycans that are components of glycolipids, glycoproteins or sulfated glycoconjugates (Jiang *et al.*, 2011 ▸; Sumida *et al.*, 2011 ▸; Manuel *et al.*, 2007 ▸; Intra *et al.*, 2008 ▸). Superimposition of the catalytic domains of *Vh*GlcNAcase and StrH in complex with NGA2B reveals that loop L7 of *Vh*GlcNacase in particular would cause steric clashes with a branched glycan, consistent with its preference for linear chitooligosaccharide chains.

## 5. Concluding remarks   

This study reports the crystal structures of a GH20 β-*N*-acetylglucosaminidase, namely *Vh*GlcNAcase, in the absence and presence of a natural ligand (GlcNAc) from the marine bacterium *V. campbellii*. *Vh*GlcNAcase contains three domains: a carbohydrate-binding domain (CBD), an α+β domain and a conserved (β/α)_8_ TIM-barrel catalytic domain. Size-exclusion chromatography confirmed that *Vh*GlcNAcase is a monomeric enzyme in solution, with an apparent MW of 74 kDa. In the complex with product, one GlcNAc was found at subsite −1 of the highly negatively charged catalytic pocket. Binding of GlcNAc induces local conformational changes around the −1 subsite, where the sugar makes contacts with polar side-chain residues through a network of hydrogen bonds and stacks against the side chain of Trp582. Site-directed mutagenesis and kinetic analysis suggest that the acidic pair Asp437 and Glu438 play an important role in catalysis. Docking of *Vh*GlcNAcase with a branched GlcNAc-derived glycan reveals severe steric clashes between the branched sugar and the active-site surface, consistent with the fact that *Vh*GlcNAcase prefers chitin-derived chitooligo­saccharides as natural substrates. The structural insight into *Vh*GlcNAcase provides a further understanding of how *Vibrio* bacteria can thrive in marine ecosystems using chitin as their sole carbon source. From a biotechnological point of view, chitinases and GlcNAcases from *Vibrio* species are chitin-degrading enzymes that are naturally responsible for the recycling of chitin, and a one-pot reaction of these two enzymes could act as a powerful biocatalyst for the complete conversion of chitin biomass into small sugar products that can be used as starting materials for further chemical modifications or for the organic synthesis of highly compatible chitin-derived functional biomaterials.

## Supplementary Material

PDB reference: *Vh*GlcNAcase, wild type, apo, 6ezr

PDB reference: wild type, complex with GlcNAc, 6ezs

PDB reference: D437A mutant, apo, 6ezt

## Figures and Tables

**Table 1:** Primers used for mutagenesis

Mutation	Oligonucleotide sequence†
D303A	
Forward	5′-CATTGGCATCTCACTGCGGATGAAGGCTGGCGTG-3′
Reverse	5′-CACGCCAGCCTTCATCCGCAGTGAGATGCCAATG-3′
D303N	
Forward	5′-CATTGGCATCTCACTAACGATGAAGGCTGGCGTG-3′
Reverse	5′-CACGCCAGCCTTCATCGTTAGTGAGATGCCAATG-3′
H373A	
Forward	5′-GAAATTGATGTACCTGGTGCGTGCCGCGCCGCAATTAAG-3′
Reverse	5′-CTTAATTGCGGCGCGGCACGCACCAGGTACATCAATTTC-3′
D437A	
Forward	5′-GTTCACATTGGCGCGGCGGAAGTGCCTAACGGC-3′
Reverse	5′-GCCGTTAGGCACTTCCGCCGCGCCAATGTGAAC-3′
D437N	
Forward	5′-GTTCACATTGGCGCGAACGAAGTGCCTAACGGC-3′
Reverse	5′-GCCGTTAGGCACTTCGTTCGCGCCAATGTGAAC-3′
E438Q	
Forward	5′-GTTCACATTGGCGCGGACCAGGTGCCTAACGGCGTGTG-3′
Reverse	5′-CACACGCCGTTAGGCACCTGGTCCGCGCCAATGTGAAC-3′
E438A	
Forward	5′-CACATTGGCGCGGACGCGGTGCCTAACGGCGTGTG-3′
Reverse	5′-CACACGCCGTTAGGCACCGCGTCCGCGCCAATGTG-3′
D532A	
Forward	5′-CAAACTACTTATTTGGCGATGACCCAAGACTACGC-3′
Reverse	5′-GCGTAGTCTTGGGTCATCGCCAAATAAGTAGTTTG-3′
E584A	
Forward	5′-CCGCTCTATGGTGCGCGATCATCAACAACCC-3′
Reverse	5′-GGGTTGTTGATGATCGCGCACCATAGAGCGG-3′

† Underlined sequences indicate mutated codons.

**Table 2:** Data-collection and refinement statistics Values in parentheses are for the outer resolution shell.

	Apo wild type (PDB entry 6ezr)	Wild type complexed with GlcNAc (PDB entry 6ezs)	Apo D437A (PDB entry 6ezt)
Data collection			
Wavelength (Å)	0.9998	0.9789	0.9789
Space group	*P*2_1_	*P*2_1_	*P*2_1_
*a*, *b*, *c* (Å)	90.2, 130.7, 98.5	91.3, 129.6, 100.0	89.4, 129.2, 98.4
α, β, γ (°)	90.0, 113.0, 90.0	90.0, 114.4, 90.0	90.0, 112.2, 90.0
Resolution range (Å)	48.55–2.37 (2.46–2.37)	48.02–2.50 (2.59–2.50)	48.42–2.60 (2.69–2.60)
*R*_merge_	0.146 (1.087)	0.118 (0.533)	0.125 (0.775)
〈*I*/σ(*I*)〉	9.93 (2.21)	9.71 (3.17)	9.70 (2.18)
CC_1/2_ (%)	99.4 (84.1)	99.2 (85.1)	99.3 (86.5)
Completeness (%)	99.6 (99.2)	99.5 (99.9)	99.9 (99.9)
Multiplicity	6.8 (7.1)	4.7 (4.5)	6.9 (7.1)
Refinement			
Resolution (Å)	2.37	2.50	2.60
Total No. of reflections	577490	342683	436477
No. of unique reflections	84782 (8365)	72949 (7297)	63607 (6320)
*R*_work_	0.2133 (0.3887)	0.2092 (0.2742)	0.2050 (0.2983)
*R*_free_	0.2543 (0.4253)	0.2554 (0.3153)	0.2389 (0.3489)
No. of atoms			
Protein	10380	10325	10300
Ligand/ion	0	44	40
Water	988	915	426
R.m.s.d.			
Bond lengths (Å)	0.009	0.009	0.007
Bond angles (°)	1.34	1.26	1.20
Wilson *B* factor (Å^2^)	44.97	33.10	56.76
Average *B* factors (Å^2^)			
Protein	55.34	36.35	64.96
Ligand	—	30.93	94.27
Solvent	53.79	34.69	56.34
Ramachandran plot			
Favored regions (%)	97.33	98.51	97.49
Allowed region (%)	2.43	1.33	2.43
Outlier regions (%)	0.24	0.16	0.08
Rotamer outliers (%)	2.6	2.1	1.7
Clashscore	1.96	2.74	1.77

**Table 3:** A summary of direct ligand–enzyme interactions obtained by *LIGPLOT* analysis Hydrogen bonds and van der Waals interactions were set at a 3.0 Å distance. The underlined amino-acid residues form hydrogen bonds, while residues shown in bold make hydrophobic interactions with the corresponding ligands.

Subsite	GH20 glycoside hydrolase			
	*Vh*GlcNAcase + GlcNAc (PDB entry 6ezs)	*Sp*HEX + GlcNAc (PDB entry 1m01)	*Sm*Chb + (GlcNAc)_2_ (PDB entry 1qbb)	*Of*HEX + TMG-chitotriomycin (PDB entry 3vtr)
−1	Arg274, **Trp487**, **Trp505**, Tyr530, Asp532, **Trp582**, Glu584	Arg162, Asp313, Glu314, **Trp344**, **Trp361**, **Tyr393**, Asp395, **Trp442**, Glu444	Arg349, Asp539, Glu540, **Trp616**, **Trp639**, **Tyr669**, Asp671, **Phe672**, **Trp737**, Glu739	Arg220, Asp249, Asp367, **Trp448**, Trp490, **Trp524**, Glu526
+1	**Trp546**	Glu314, **Trp408**	Glu540, **Trp685**	**Trp448**, **Trp483**, **Trp490**, **Trp524**
+2	—	—	—	—
+3	—	—	—	—

**Table 4:** Steady-state kinetic parameters for *p*NP-GlcNAc hydrolysis by *Vh*GlcNAcase variants

*Vh*GlcNAcase	*K*_m_ (µ*M*)	*k*_cat_ (s^−1^)	*k*_cat_/*K*_m_ (× 10^3^ s^−1^ *M* ^−1^)	Fold decrease in catalytic efficiency
Wild type	92 ± 6	28 ± 0.6	304 (100%)	—
D303A	302 ± 28	1.4 ± 0.07	4.6 (1.6%)	66
D303N	325 ± 30	1.8 ± 0.08	5.5 (1.8%)	55
H373A	307 ± 31	1.1 ± 0.06	3.6 (1.2%)	85
D437A	390 ± 32	0.14 ± 0.006	0.4 (0.12%)	848
D437N	339 ± 47	0.02 ± 0.001	0.1 (0.01%)	5159
E438A	128 ± 16	0.11 ± 0.005	0.9 (0.28%)	354
E438Q	120 ± 13	0.13 ± 0.005	1.1 (0.35%)	281
D532A	153 ± 9	1.6 ± 0.03	10.5 (3.32%)	29
E584A	178 ± 20	18 ± 0.7	101 (32.0%)	3

## References

[bb1] Bassler, B. L., Yu, C., Lee, Y. C. & Roseman, S. (1991). *J. Biol. Chem.* **266**, 24276–24286. 1761533

[bb13] Battye, T. G. G., Kontogiannis, L., Johnson, O., Powell, H. R. & Leslie, A. G. W. (2011). *Acta Cryst.* D**67**, 271–281. 10.1107/S0907444910048675 PMC3069742 21460445

[bb16] Chen, V. B., Arendall, W. B., Headd, J. J., Keedy, D. A., Immormino, R. M., Kapral, G. J., Murray, L. W., Richardson, J. S. & Richardson, D. C. (2010). *Acta Cryst.* D**66**, 12–21. 10.1107/S0907444909042073 PMC2803126 20057044

[bb20] Cremer, D. & Pople, J. A. (1975). *J. Am. Chem. Soc.* **97**, 1354–1358.

[bb22] DeLano, W. L. (2002). *PyMOL*. http://www.pymol.org.

[bb24] Emsley, P., Lohkamp, B., Scott, W. G. & Cowtan, K. (2010). *Acta Cryst.* D**66**, 486–501. 10.1107/S0907444910007493 PMC2852313 20383002

[bb27] Federico, A., Palmeri, S., Malandrini, A., Fabrizi, G., Mondelli, M. & Guazzi, G. C. (1991). *Dev. Neurosci.* **13**, 280–287. 10.1159/000112174 1840098

[bb29] Gill, S. C. & von Hippel, P. H. (1989). *Anal. Biochem.* **182**, 319–326. 10.1016/0003-2697(89)90602-7 2610349

[bb31] Holm, L. & Sander, C. (1993). *J. Mol. Biol.* **233**, 123–138. 10.1006/jmbi.1993.1489 8377180

[bb34] Intra, J., Pavesi, G. & Horner, D. S. (2008). *BMC Evol. Biol.* **8**, 214. 10.1186/1471-2148-8-214 PMC2492878 18647384

[bb37] Jeffrey, G. A. & Yates, J. H. (1979). *Carbohydr. Res.* **74**, 319–322.

[bb38] Jiang, Y.-L., Yu, W.-L., Zhang, J.-W., Frolet, C., Di Guilmi, A. M., Zhou, C.-Z., Vernet, T. & Chen, Y. (2011). *J. Biol. Chem.* **286**, 43004–43012. 10.1074/jbc.M111.256578 PMC3234876 22013074

[bb41] Kabsch, W. (2010). *Acta Cryst.* D**66**, 125–132. 10.1107/S0907444909047337 PMC2815665 20124692

[bb43] Keyhani, N. O. & Roseman, S. (1999). *Biochim. Biophys. Acta*, **1473**, 108–122. 10.1016/s0304-4165(99)00172-5 10580132

[bb45] King, S. J. (2010). *Mol. Oral Microbiol.* **25**, 15–24. 10.1111/j.2041-1014.2009.00564.x 20331791

[bb47] Krissinel, E. & Henrick, K. (2007). *J. Mol. Biol.* **372**, 774–797. 10.1016/j.jmb.2007.05.022 17681537

[bb49] Laskowski, R. A., Moss, D. S. & Thornton, J. M. (1993). *J. Mol. Biol.* **231**, 1049–1067. 10.1006/jmbi.1993.1351 8515464

[bb51] Laskowski, R. A. & Swindells, M. B. (2011). *J. Chem. Inf. Model.* **51**, 2778–2786. 10.1021/ci200227u 21919503

[bb53] Lemieux, M. J., Mark, B. L., Cherney, M. M., Withers, S. G., Mahuran, D. J. & James, M. N. G. (2006). *J. Mol. Biol.* **359**, 913–929. 10.1016/j.jmb.2006.04.004 PMC2910082 16698036

[bb55] Li, B., Li, H., Hu, C.-W. & Jiang, J. (2017). *Nat. Commun.* **8**, 666. 10.1038/s41467-017-00865-1 PMC5610315 28939839

[bb57] Li, X. & Roseman, S. (2004). *Proc. Natl Acad. Sci. USA*, **101**, 627–631. 10.1073/pnas.0307645100 PMC327198 14699052

[bb59] Liu, T., Zhang, H., Liu, F., Wu, Q., Shen, X. & Yang, Q. (2011). *J. Biol. Chem.* **286**, 4049–4058. 10.1074/jbc.M110.184796 PMC3039403 21106526

[bb61] Lombard, V., Golaconda Ramulu, H., Drula, E., Coutinho, P. M. & Henrissat, B. (2014). *Nucleic Acids Res.* **42**, D490–D495. 10.1093/nar/gkt1178 PMC3965031 24270786

[bb63] Maier, T., Strater, N., Schuette, C. G., Klingenstein, R., Sandhoff, K. & Saenger, W. (2003). *J. Mol. Biol.* **328**, 669–681. 10.1016/s0022-2836(03)00311-5 12706724

[bb65] Manuel, S. G., Ragunath, C., Sait, H. B., Izano, E. A., Kaplan, J. B. & Ramasubbu, N. (2007). *FEBS J.* **274**, 5987–5999. 10.1111/j.1742-4658.2007.06121.x 17949435

[bb67] Mark, B. L., Mahuran, D. J., Cherney, M. M., Zhao, D., Knapp, S. & James, M. N. G. (2003). *J. Mol. Biol.* **327**, 1093–1109. 10.1016/s0022-2836(03)00216-x PMC2910754 12662933

[bb69] Mark, B. L., Vocadlo, D. J., Knapp, S., Triggs-Raine, B. L., Withers, S. J. & James, M. N. G. (2001). *J. Biol. Chem.* **276**, 10330–10337. 10.1074/jbc.M011067200 11124970

[bb71] McCoy, A. J., Grosse-Kunstleve, R. W., Adams, P. D., Winn, M. D., Storoni, L. C. & Read, R. J. (2007). *J. Appl. Cryst.* **40**, 658–674. 10.1107/S0021889807021206 PMC2483472 19461840

[bb72] Meekrathok, P., Bürger, M., Porfetye, A. T., Vetter, I. R. & Suginta, W. (2015). *Acta Cryst.* F**71**, 427–433. 10.1107/S2053230X1500415X PMC4388178 25849504

[bb75] Meekrathok, P., Stubbs, K. A., Aunkham, A., Kaewmaneewat, A., Kardkuntod, A., Bulmer, D. M., van den Berg, B. & Suginta, W. (2020). *FEBS J.* **287**, 4982–4995. 10.1111/febs.15283 32145141

[bb77] Meekrathok, P. & Suginta, W. (2016). *PLoS One*, **11**, e0149228. 10.1371/journal.pone.0149228 PMC4752478 26870945

[bb79] Murshudov, G. N., Skubák, P., Lebedev, A. A., Pannu, N. S., Steiner, R. A., Nicholls, R. A., Winn, M. D., Long, F. & Vagin, A. A. (2011). *Acta Cryst.* D**67**, 355–367. 10.1107/S0907444911001314 PMC3069751 21460454

[bb80] Myerowitz, R. (1997). *Hum. Mutat.* **9**, 195–208. 10.1002/(SICI)1098-1004(1997)9:3<195::AID-HUMU1>3.0.CO;2-7 9090523

[bb85] Park, J. K., Keyhani, N. O. & Roseman, S. (2000). *J. Biol. Chem.* **275**, 33077–33083. 10.1074/jbc.M001042200 10913116

[bb88] Sandhoff, K. & Kolter, T. (1998). *Acta Biochim. Pol.* **45**, 373–384. 9821868

[bb90] Sirimontree, P., Fukamizo, T. & Suginta, W. (2016). *J. Biochem.* **159**, 191–200. 10.1093/jb/mvv087 PMC4892774 26330565

[bb93] Songsiriritthigul, C., Pantoom, S., Aguda, A. H., Robinson, R. C. & Suginta, W. (2008). *J. Struct. Biol.* **162**, 491–499. 10.1016/j.jsb.2008.03.008 18467126

[bb95] Soto-Gil, R. W. & Zyskind, J. W. (1984). *Chitin, Chitosan and Related Enzymes*, edited by J. P. Zikakis, pp. 209–223. New York: Academic Press.

[bb97] Suginta, W., Chuenark, D., Mizuhara, M. & Fukamizo, T. (2010). *BMC Biochem.* **11**, 40. 10.1186/1471-2091-11-40 PMC2955587 20920218

[bb99] Suginta, W., Chumjan, W., Mahendran, K. R., Janning, P., Schulte, A. & Winterhalter, M. (2013). *PLoS One*, **8**, e55126. 10.1371/journal.pone.0055126 PMC3558487 23383078

[bb101] Suginta, W., Chumjan, W., Mahendran, K. R., Schulte, A. & Winterhalter, M. (2013). *J. Biol. Chem.* **288**, 11038–11046. 10.1074/jbc.M113.454108 PMC3630896 23447539

[bb102] Suginta, W., Vongsuwan, A., Songsiriritthigul, C., Prinz, H., Estibeiro, P., Duncan, R. R., Svasti, J. & Fothergill-Gilmore, L. A. (2004). *Arch. Biochem. Biophys.* **424**, 171–180. 10.1016/j.abb.2004.01.017 15047189

[bb104] Suginta, W., Vongsuwan, A., Songsiriritthigul, C., Svasti, J. & Prinz, H. (2005). *FEBS J.* **272**, 3376–3386. 10.1111/j.1742-4658.2005.04753.x 15978043

[bb106] Sumida, T., Fujimoto, K. & Ito, M. (2011). *J. Biol. Chem.* **286**, 14065–14072. 10.1074/jbc.M110.182592 PMC3077607 21297160

[bb108] Sumida, T., Ishii, R., Yanagisawa, T., Yokoyama, S. & Ito, M. (2009). *J. Mol. Biol.* **392**, 87–99. 10.1016/j.jmb.2009.06.025 19524595

[bb110] Tayyab, S., Qamar, S. & Islam, M. (1991). *Biochem. Educ.* **19**, 149–152.

[bb112] Tews, I., Perrakis, A., Oppenheim, A., Dauter, Z., Wilson, K. S. & Vorgias, C. E. (1996). *Nat. Struct. Mol. Biol.* **3**, 638–648. 10.1038/nsb0796-638 8673609

[bb114] Thi, N. N., Offen, W. A., Shareck, F., Davies, G. J. & Doucet, N. (2014). *Biochemistry*, **53**, 1789–1800. 10.1021/bi401697j 24559145

[bb116] Val-Cid, C., Biarnés, X., Faijes, M. & Planas, A. (2015). *PLoS One*, **10**, e0128075. 10.1371/journal.pone.0128075 PMC4449183 26024355

[bb118] Vocadlo, D. J. & Withers, S. G. (2005). *Biochemistry*, **44**, 12809–12818. 10.1021/bi051121k 16171396

[bb119] Williams, S. J., Mark, B. L., Vocadlo, D. J., James, M. N. G. & Withers, S. G. (2002). *J. Biol. Chem.* **277**, 40055–40065. 10.1074/jbc.M206481200 12171933

[bb18] Winn, M. D., Ballard, C. C., Cowtan, K. D., Dodson, E. J., Emsley, P., Evans, P. R., Keegan, R. M., Krissinel, E. B., Leslie, A. G. W., McCoy, A., McNicholas, S. J., Murshudov, G. N., Pannu, N. S., Potterton, E. A., Powell, H. R., Read, R. J., Vagin, A. & Wilson, K. S. (2011). *Acta Cryst.* D**67**, 235–242. 10.1107/S0907444910045749 PMC3069738 21460441

[bb122] Yu, C., Lee, A. M., Bassler, B. L. & Roseman, S. (1991). *J. Biol. Chem.* **266**, 24260–24267. 1761531

